# HUGO Gene Nomenclature Committee (HGNC) recommendations for the designation of gene fusions

**DOI:** 10.1038/s41375-021-01436-6

**Published:** 2021-10-06

**Authors:** Elspeth A. Bruford, Cristina R. Antonescu, Andrew J. Carroll, Arul Chinnaiyan, Ian A. Cree, Nicholas C. P. Cross, Raymond Dalgleish, Robert Peter Gale, Christine J. Harrison, Rosalind J. Hastings, Jean-Loup Huret, Bertil Johansson, Michelle Le Beau, Cristina Mecucci, Fredrik Mertens, Roel Verhaak, Felix Mitelman

**Affiliations:** 1 HUGO Gene Nomenclature Committee (HGNC), European Molecular Biology Laboratory, European Bioinformatics Institute, Wellcome Genome Campus, Hinxton, UK; 2 Department of Haematology, University of Cambridge School of Clinical Medicine, Cambridge, UK; 3 Department of Pathology, Memorial Sloan Kettering Cancer Center, New York, NY USA; 4 Department of Genetics, University of Alabama at Birmingham, Birmingham, AL USA; 5 University of Michigan Medical School, Ann Arbor, MI USA; 6 Howard Hughes Medical Institute, Chevy Chase, MD USA; 7 International Agency for Research on Cancer, World Health Organization, Lyon, France; 8 Faculty of Medicine, University of Southampton, Southampton, UK; 9 Wessex Regional Genetics Laboratory, Salisbury NHS Foundation Trust, Salisbury, Wiltshire UK; 10 Department of Genetics and Genome Biology, University of Leicester, Leicester, UK; 11 Centre for Haematology Research, Department of Immunology and Inflammation, Imperial College London, London, UK; 12 Translational and Clinical Research Institute, Newcastle University Centre for Cancer, Newcastle upon Tyne, UK; 13 The Women’s Centre, John Radcliffe Hospital, Oxford University Hospitals Foundation Trust, Oxford, UK; 14 10 rue des Treilles, Masseuil, Quinçay France; 15 Division of Clinical Genetics, Department of Laboratory Medicine, Lund University, Lund, Sweden; 16 Comprehensive Cancer Center, University of Chicago, Chicago, IL USA; 17 Department of Medicine and Surgery, University of Perugia, Perugia, Italy; 18 Jackson Laboratory for Genomic Medicine, Farmington, CT USA; 19 Department of Neurosurgery, Amsterdam University Medical Center, Amsterdam, Netherlands

**Keywords:** Cancer genetics, Genetic translocation, Cancer genetics, Cancer genetics, Cancer genomics

## Abstract

Gene fusions have been discussed in the scientific literature since they were first detected in cancer cells in the early 1980s. There is currently no standardized way to denote the genes involved in fusions, but in the majority of publications the gene symbols in question are listed either separated by a hyphen (-) or by a forward slash (/). Both types of designation suffer from important shortcomings. HGNC has worked with the scientific community to determine a new, instantly recognizable and unique separator—a double colon (::)—to be used in the description of fusion genes, and advocates its usage in all databases and articles describing gene fusions.

## Brief historical background of gene fusions

**Fig. 1:**
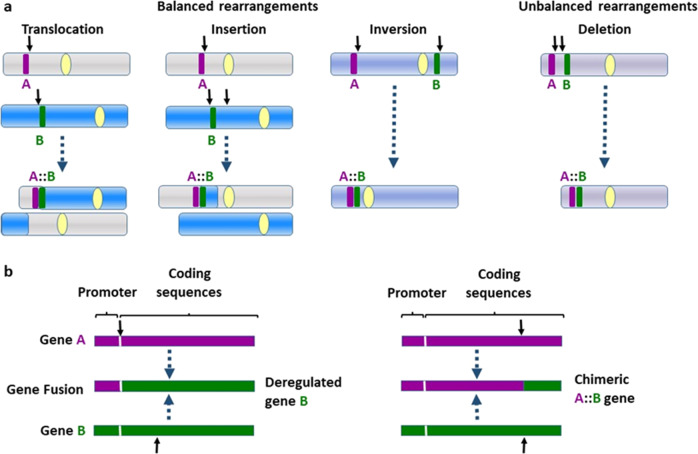
The chromosomal basis of gene fusions. **a** Gene fusions may originate through balanced and unbalanced chromosome rearrangements. Balanced changes comprise translocations (the transfer of chromosome segments between chromosomes), insertions (a chromosome segment in a new interstitial position in the same or another chromosome) and inversions (rotation of a chromosome segment by 180°); an example of an unbalanced change is the deletion of an interstitial chromosomal segment. Small arrows indicate breakpoints, and large arrows indicate the resulting rearranged chromosomes. A and B signify affected genes. Note that a reciprocal gene fusion may be generated on the partner derivative chromosome as a result of a reciprocal translocation, but this is not shown. **b** Both balanced and unbalanced aberrations may lead to the deregulation of either gene A or gene B by the juxtaposition of the coding sequences with the regulatory sequences of the other gene, or to the creation of a chimeric gene through the fusion of parts of both genes.

Technical developments at the end of the 1970s enabled the identification of genes in the breakpoints of chromosome rearrangements, which in the early 1980s led to the discovery and characterization of gene fusions in neoplasia. While the products of translocation events are referred to by several terms, including fusion genes, hybrid genes and chimeric genes, here we largely choose to use the term fusion genes as this is most widely used in this context. Analyses of the recurrent balanced translocations in Burkitt lymphoma (BL) and chronic myeloid leukemia (CML) proved particularly pivotal. The picture to emerge was that reciprocal translocations exert their effects by one of two alternative mechanisms: deregulation, usually resulting in the overexpression of a seemingly normal gene in one of the breakpoints, or the creation of a hybrid, chimeric gene through fusion of parts of two genes, one in each breakpoint [1] (see Fig. 1).

BL provided the first conclusive evidence for the deregulation mechanism. This tumor type was found to harbor one of three translocations: t(8;14)(q24;q32), t(2;8)(p11;q24) or t(8;22)(q24;q11). In all three, the breakpoints in chromosome 8 were found to be within or adjacent to the *MYC* oncogene (8q24), and the other breakpoint in an immunoglobulin gene, encoding the heavy chain (*IGH* in 14q32) or the kappa (*IGK* in 2p11) or lambda (*IGL* in 22q11) light chains [2–4]. As a consequence of these translocations, the *MYC* gene becomes transcriptionally deregulated, often overexpressed, owing to the influence of regulatory elements of the immunoglobulin genes.

The alternative mechanism, the creation of a hybrid gene, was documented at the same time in CML with the demonstration that the Philadelphia chromosome, i.e., the derivative chromosome 22 resulting from the recurrent reciprocal translocation t(9;22)(q34;q11), juxtaposed the 5′ part of the *BCR* gene at 22q11 with the 3′ part of the *ABL1* tyrosine kinase-encoding gene from 9q34. This leads to an in-frame fusion of parts of the two genes and results in an abnormal protein, which displays increased tyrosine kinase activity [5–8] (see Fig. 1).

These and similar molecular insights into how cancer-specific chromosomal abnormalities act pathogenetically sparked an enormous interest in cytogenetics as a powerful means to pinpoint the locations of genes important in tumorigenesis, and an impressive amount of information has been accumulated through these efforts. Almost 1000 gene fusions have been found by genomic characterization of breakpoints in cytogenetically identified aberrations, balanced as well as unbalanced, in various leukemias, lymphomas, and solid tumors. The accumulated data have shown that the consequences of practically all gene fusions are in principle the same as those originally elucidated in BL and CML, i.e., deregulation of a seemingly normal gene or the creation of a hybrid gene. However, not all gene fusions have translational consequences, and some could result in gene inactivation [1, 9, 10].

The advent of massively parallel sequencing (MPS) has recently provided a radically new means to identify fusions at the DNA or RNA levels without any prior information on the cytogenetic features of the neoplastic cells. The results of such unbiased gene fusion detection efforts during the last decade have dramatically changed the gene fusion landscape. More than 30,000 gene fusions, the great majority involving previously unsuspected genes, have now been identified through deep sequencing in a wide variety of neoplasms and these are reported in a number of online resources, e.g., https://mitelmandatabase.isb-cgc.org/, http://atlasgeneticsoncology.org/, https://cancer.sanger.ac.uk/cosmic, https://ccsm.uth.edu/FusionGDB/, http://www.kobic.re.kr/chimerdb/, https://tumorfusions.org/. A major challenge will be to verify by functional studies which of the alleged gene fusions are pathogenetically important in carcinogenesis, and which are either secondary progressional changes or non-consequential “noise” abnormalities, e.g., by-products of the genetic instability that characterizes many cancer cells. It is important to note that while gene fusions are a hallmark of neoplasia, they can also occur in heritable disorders such as the formation of the Lepore and anti-Lepore haemoglobins from the *HBD* and *HBB* genes [11].

There has never been a generally recommended, standardized way to denote gene fusions. Instead, multiple notations have been used with varying popularity over time, though the most common designation is SYMBOL-SYMBOL followed by SYMBOL/SYMBOL, both of which we critique below.

## Problems with the nomenclature used to describe gene fusions

The SYMBOL-SYMBOL notation, e.g., *BCR*-*ABL1*, to denote fusion genes has three important shortcomings:

(1) The HGNC has approved the use of the hyphen separator, in collaboration with all contributing genome annotation groups involved in the Consensus Coding Sequence (CCDS) Project [12], for denoting readthrough transcripts, e.g., *INS-IGF2*.

(2) A hyphen is often used in the literature to denote members of a complex, e.g., MRE11-NBN, MRE11-RAD50-NBN.

(3) There are also specific groups of approved gene symbols containing hyphens as separators within the symbol, e.g., *TRX-CAT1-2*.

Hence, it is difficult to search specifically for gene fusions in databases and in the literature using the hyphen symbol.

The SYMBOL/SYMBOL notation, e.g., *BCR/ABL1*, has at least four major disadvantages:

(1) The forward slash is an accepted symbol in the established cytogenetic International System for Human Cytogenomic Nomenclature (ISCN) to denote different clones, both constitutionally (mosaicism) and in cancer cells; the Human Genome Variation Society (HGVS) guidelines (https://varnomen.hgvs.org/recommendations/general/) also use a forward slash to indicate mosaicism [13].

(2) The forward slash is often used in the literature in place of “either/or”, e.g., *BRCA1*/*2*, and to denote involvement of alternative genes in a fusion, e.g., “*SS18-SSX1*/*SSX2”*.

(3) Pathway and complex descriptions use this character, e.g., *RAS*/*RAF*/*MAPK*.

(4) Commercial dual fusion fluorescence in situ hybridization translocation probes (CE marked) also use a forward slash to indicate the two probe sets used, e.g., *BCR*/*ABL1*.

In view of the considerations listed above, and hence the clear need for a standardized and unique way to denote gene fusions, the HGNC concluded that an alternative needed to be sought to replace the use of either a hyphen (-) or a forward slash (/).

## Recommended new nomenclature to describe gene fusions

After careful deliberation, and consultations with experts in the field, HGNC recommends that a new separator—a double colon (::)—be used in describing gene fusions, e.g., *BCR*::*ABL1*. The double colon (::) has several important advantages:

First, it follows the long-standing recommendation of the internationally accepted ISCN cytogenetic nomenclature in which a single colon (:) is used to indicate a chromosome break and a double colon (::) to denote *break and reunion* [14]. The:: separator thus nicely reflects the principal mode of origin of most fusion genes. We are aware that fusion transcripts may occasionally originate at the RNA level through *cis*- or *trans*-splicing without a genomic breakage and reunion correlate, but we deem it unnecessary to create a different nomenclature system for such events, especially since the HGVS already recommends using the double colon to describe RNA fusion transcripts (https://varnomen.hgvs.org/recommendations/general/).

Secondly, it is instantly recognizable and creates a unique symbol in the existing gene nomenclature, and hence is easily searchable in databases and in the literature.

Thirdly, different gene fusions found in different single cells or in separate clones within the same tumor will be easily recognizable, i.e., SYMBOL::SYMBOL/SYMBOL::SYMBOL.

## Specific recommendations when describing gene fusions

In line with established HGNC recommendations [15], genes involved in fusions should be designated by their HGNC approved gene symbols written in italics, whereas proteins are not italicized, e.g., *BCR*::*ABL1* denotes a fusion of the *BCR* and *ABL1* genes, while BCR::ABL1 designates the corresponding protein product. By convention, fusion transcripts identified at the RNA level, e.g., by RNA-seq, are designated as genes, i.e., in italics. The double colon (::) should be used for all types of gene fusions, i.e., both those giving rise to a hybrid, chimeric gene (*BCR*::*ABL1*) and those where regulatory elements from one gene deregulate a partner gene (*IGH*::*MYC*).

In accordance with established practice in designating gene fusions, the 5′ partner gene should always be listed first in the description of a fusion gene, i.e., before the double colon, irrespective of chromosomal location or the orientation of the gene. Thus, in the *BCR*::*ABL1* fusion gene—the outcome of the translocation t(9;22)(q34.1;q11.2)—the *BCR* gene in chromosome 22 is the 5′ gene, the *ABL1* gene from chromosome 9 is the 3′ gene.

In tables in scientific articles and in databases presenting gene fusions, the two genes are often designated either as “Gene A” and “Gene B” or “Gene 1” and “Gene 2”. Thus, it is not explicitly stated that “Gene A” or “Gene 1” represents the 5′ gene although this is usually the case. To avoid ambiguous interpretations, HGNC recommends that 5′ and 3′ genes be clearly indicated in tables showing gene fusions. In fusions giving rise to a deregulated gene the regulatory or enhancer element should be listed first, whenever known.

If one of the genes in a fusion is unknown this may be indicated by either a question mark (?) or by the chromosomal band where the breakpoint is located, following ISCN convention. If one of the breakpoints lies in an intergenic region and the genomic coordinate of that breakpoint is known, this can be denoted in an abbreviated format for publication as chr#:g.coordinate number. For example, *ABL1*::? denotes a fusion between *ABL1* and an unknown gene, 6q25::*ABL1* a fusion between an unknown gene located in chromosome band 6q25 and *ABL1*, and *ABL1*::chr11.g:1850000 a fusion between *ABL1* and a breakpoint at nucleotide 1,850,000 on chromosome 11. In the first and third examples the unknown gene and intergenic region are the 3′ partners, and in the second example the unknown gene is the 5′ partner. Full ISCN [14] (https://iscn.karger.com/) or HGVS (https://varnomen.hgvs.org/recommendations/DNA/variant/complex/) nomenclature should be used for formal reporting.

Note that HGNC always advocate listing a stable gene ID, ideally an HGNC ID, when referencing genes in publications. We do not recommend that the IDs be included in the fusion notation, but rather in the accompanying text, e.g., a gene fusion involving *BCR*(HGNC:1014) and *ABL1*(HGNC:76) is denoted as *BCR*::*ABL1*.

## Conclusions

There has long been a need for a unique, standardized and easily recognizable way to symbolize gene fusion events consistently, both in the literature and in databases. Following consultation with experts in the field of gene fusions, HGNC recommends the use of the separator “::”, a double colon, between approved gene symbols, to designate the genes involved in gene fusion events, e.g., *BCR*::*ABL1*. This recommendation is further endorsed by all the authors of this manuscript, by the HGVS and the ISCN, and by the following resources: the WHO Classification of Tumors, COSMIC, OMIM, Atlas of Genetics and Cytogenetics in Oncology and Haematology, Mitelman Database of Chromosome Aberrations and Gene Fusions in Cancer, and the Tumor Fusion Gene Data Portal. We urge all readers to use and publicize this form of notation for describing gene fusions in all future communications to avoid confusion. We recognize that this is a newly established recommendation that could be further developed in due course, and welcome feedback from the community (via the HGNC website, www.genenames.org).

## References

[1] Mertens F, Johansson B, Fioretos T, Mitelman F (2015). The emerging complexity of gene fusions in cancer. Nat Rev Cancer.

[2] Dalla-Favera R, Bregni M, Erikson J, Patterson D, Gallo RC, Croce CM (1982). Human c-*myc onc* gene is located on the region of chromosome 8 that is translocated in Burkitt lymphoma cells. Proc Natl Acad Sci USA.

[3] Taub R, Kirsch I, Morton C, Lenoir G, Swan D, Tronick S (1982). Translocation of the c-*myc* gene into the immunoglobulin heavy chain locus in human Burkitt lymphoma and murine plasmacytoma cells. Proc Natl Acad Sci USA.

[4] Erikson J, Nishikura K, ar-Rushdi A, Finan J, Emanuel B, Lenoir G (1983). Translocation of an immunoglobulin κ locus to a region 3’ of an unrearranged c-*myc* oncogene enhances c-*myc* transcription. Proc Natl Acad Sci USA.

[5] Heisterkamp N, Stam K, Groffen J, de Klein A, Grosveld G (1985). Structural organization of the *bcr* gene and its role in the Ph’ translocation. Nature.

[6] Shtivelman E, Lifshitz B, Gale RP, Canaani E (1985). Fused transcript of *abl* and *bcr* genes in chronic myelogenous leukaemia. Nature.

[7] Ben-Neriah Y, Daley GQ, Mes-Masson AM, Witte ON, Baltimore D (1986). The chronic myelogenous leukemia-specific P210 protein is the product of the bcr/abl hybrid gene. Science.

[8] Fainstein E, Marcelle C, Rosner A, Canaani E, Gale RP, Dreazen O (1987). A new fused transcript in Philadelphia chromosome positive acute lymphocytic leukaemia. Nature.

[9] Kumar-Sinha C, Kalyana-Sundaram S, Chinnaiyan AM (2015). Landscape of gene fusions in epithelial cancers: seq and ye shall find. Genome Med.

[10] Rowley JD, Le Beau MM, Rabbitts TH, editors. Chromosomal Translocations and Genome Rearrangements in Cancer. New York: Springer; 2015.

[11] Efremov GD (1978). Hemoglobins Lepore and anti-Lepore. Hemoglobin.

[12] Pujar S, O’Leary NA, Farrell CM, Loveland JE, Mudge JM, Wallin C (2018). Consensus coding sequence (CCDS) database: a standardized set of human and mouse protein-coding regions supported by expert curation. Nucleic Acids Res.

[13] den Dunnen JT, Dalgleish R, Maglott DR, Hart RK, Greenblatt MS, McGowan-Jordan J (2016). HGVS recommendations for the description of sequence variants: 2016 Update. Hum Mutat.

[14] McGowan-Jordan J, Hastings R, Moore S, editors. An International System for Human Cytogenomic Nomenclature. Cytogenet Genome Res. 2020;160:341–503. 10.1159/000516655 34407535

[15] Bruford EA, Braschi B, Denny P, Jones TEM, Seal RL, Tweedie S (2020). Guidelines for human gene nomenclature. Nat Genet.

